# The complete mitochondrial genome of *Montipora efflorescens* (Scleractinia: Acroporidae)

**DOI:** 10.1080/23802359.2019.1667278

**Published:** 2020-02-06

**Authors:** In-Young Cho, Sung-Jin Hwang, Keun-Yong Kim, Chang Ho Yi, Il Hun Kim, Min-Seop Kim

**Affiliations:** a National Marine Biodiversity Institute of Korea, Seocheon, Republic of Korea;; b Department of Eco-Biological Science, Woosuk University, Jincheon-eup, Republic of Korea;; c Korea Maritime and Ocean University, Busan, Republic of Korea;; d School of Biological Sciences, College of Natural Sciences, Seoul National University, Seoul, Republic of Korea

**Keywords:** Mitochondrial genome, phylogeny, *Montipora efflorescens*, Acroporidae, Scleractinian corals

## Abstract

The complete mitogenome of the Sclreractinia, *Montipora efflorescens* Bernard, 1897 was sequenced for the first time. It had 17,887 bp, with 13 protein-coding genes, and two rRNA and two tRNA genes. Composition of *M. efflorescens* mitogenome was identical to that of typical Scleractinians. In conclusion, the complete mitogenome may provide detailed information on coral phylogeny.

*Montipora efflorescens* Bernard, 1897 is one of the reef builder species in marine ecology. It is a zooxanthellate scleractinian coral belonging to the family Acroporidae. Although it is widely distributed, spanning the Indian and Pacific Oceans, it is absent in the Atlantic (Van Oppen et al. 2004). The northern limit of distribution of *M. efflorescens* is South Korea's southern part of Jeju Island (Song 1991), and its habitat is reported to have gradually decreased worldwide (IUCN 2014; Wilkinson 2004). This species is listed in CITES (Convention on International Trade in Endangered Species of Wild Fauna and Flora) Appendix II (1990) with other Scleractinian corals.

In this study, we report the complete mitochondrial genome of *M*. *efflorescens*. Samples were collected from Jeju Island, Korea (33°13′40.9″N, 126°35′59.1″E) and deposited in the National Marine Biodiversity Institute of Korea (MABIK CN00079316). Genomic DNA was extracted from the polyp tissue of this voucher specimen. Complete mitochondrial DNA was sequenced using long-range PCR and primer walking method. Mitogenomic sequences of all Species belonging to the Acroporidae were retrieved from GenBank and aligned with *M*. *efflorescens* mitogenome analyzed in this study. Alignment of protein-coding genes accounted for amino-acid sequences and partitioned nucleotides into codons. Maximum-likelihood (ML) and Bayesian inference (BI) analyses were performed in RAxML 7.0.4 (Stamatakis 2006) and MrBayes 3.1.2 (Ronquist and Huelsenbeck 2003).

The complete mitochondrial genome of *M*. *efflorescens* is 17,887 bp in length (GenBank MG851914), comprising of 13 protein-coding genes (*nad5-5', nad1, cob, nad2, nad6, atp6, nad4, cox3, cox2, nad4l, nad3, nad5-5', atp8,* and *cox1*), two ribosomal RNA genes (*rrnL* and *rrnS*), and two transfer RNA genes (*trnM* and *trnW*). The composition of mitogenome was same as that of a typical scleractinian. Moreover, arrangement of mitogenome genes in *M*. *efflorescens* was the same as of those belonging to the same taxa. On the other hand, thirteen protein-coding genes used ATA and GTG as start codons, and ATA and TAG as stop codons. In order to confirm the phylogenetic position of the coral, information on 21 species of Acroporidae and two out groups were set up to determine the phylogeny.

**Figure 1.**
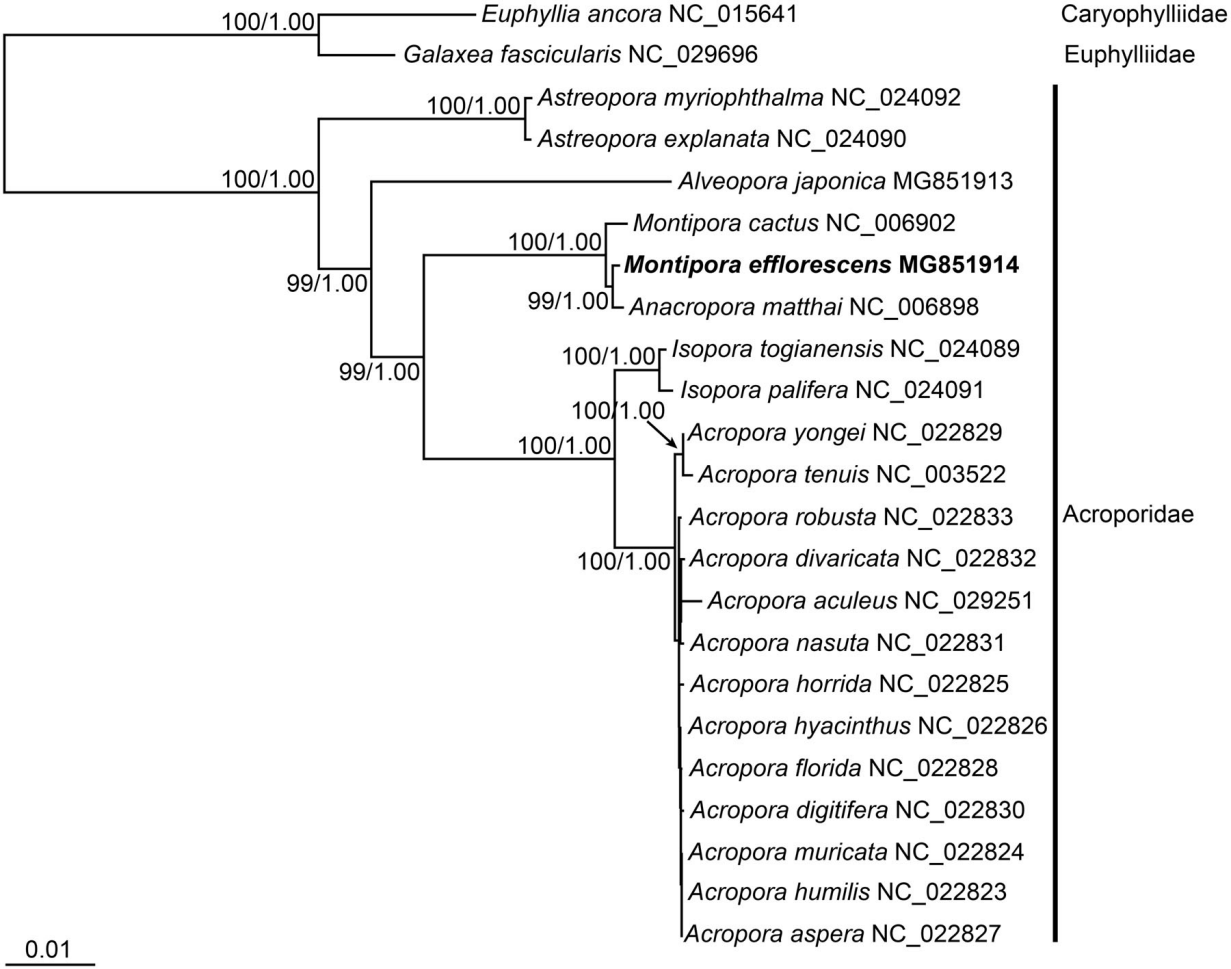
Maximum-likelihood (ML) tree inferred from the mitogenomic sequences of the species belonging to the family Acroporidae. The sequence matrix used in the phylogenetic analyses consisted of three codon positions of the protein-coding genes. Bootstrap values above 50% in the ML analysis and posterior probabilities above 0.90 in the Bayesian inference analysis are indicated at each node. Montipora efflorescens investigated in this study is shown in bold.

Results showed that corals of family Acroporidae were bound to a single clade. However, the relationship of *Anacropora matthai* Pillai and *M*. *efflorescens* was closer than *Montipora cactus* Bernard which is the same genus, suggesting the necessity of further studies in the future.
